# Prediction models of adverse outcomes following surgery and radiotherapy for breast cancer: a systematic review

**DOI:** 10.1016/j.esmorw.2026.100690

**Published:** 2026-03-10

**Authors:** H. Asfour, B. Wang, H. Zhou, A. Al Janapy, N.G. Patel, R.P. Symonds, C.J. Talbot, T. Rattay

**Affiliations:** 1 Department of Breast Surgery, University Hospitals of Leicester NHS Trust, Leicester, UK; 2 Leicester Cancer Research Centre, Department of Genetics, Genomics & Cancer Sciences, University of Leicester, Leicester, UK; 3 School of Mathematics and Computer Science, University of Leicester, Leicester, UK; 4 Department of Plastic Surgery, University Hospitals of Leicester NHS Trust, Leicester, UK

**Keywords:** prediction model, breast cancer, breast surgery, breast reconstruction, radiotherapy, adverse outcomes

## Abstract

Breast surgery and radiotherapy are the most common treatment modalities for breast cancer, although both may have side-effects that can affect quality of life. Being able to identify patients at risk of adverse outcomes would enable optimisation of individualised treatment plans to improve the experience of breast cancer survivors. A systematic review of prediction models for adverse outcomes following surgery and radiotherapy for breast cancer was conducted. PubMed, Medline, Scopus, Web of Science, and CINAHL databases were searched using relevant key words and Medical Subject Heading terms. The search yielded 5376 articles, of which 33 articles were included. Data were extracted on study design, sources of training and test/validation data, predictors, outcomes, model performance, and validation. Several prediction models for adverse outcomes following breast surgery with or without radiotherapy have been developed. For short-term side-effects, these include the American College of Surgeons National Surgical Quality Improvement Program Surgical Risk Calculator, the Breast Reconstruction Risk Assessment score, and the Breast Cancer Surgery Risk Calculator. Despite the relatively large training datasets used in the development of prediction models, they suffer from a relative lack of external validation. There is as yet no externally validated prediction model for long-term adverse outcomes, although machine learning and multiscale finite element models show promise. Overall, while significant advancements have been made in developing these prediction models, the majority are not yet ready for widespread clinical implementation. This systematic review also highlights a lack of prediction models for long-term side-effects and more complex outcomes, suggesting areas for future research.

## Highlights

• A systematic review of prediction models for side-effects from breast cancer therapy was conducted.
• The majority of models lack external validation, limiting their clinical use.
• ML-based models show promise for complex endpoints.
• Few models predict long-term effects like breast appearance and QoL.
• Future research should prioritise external validation across diverse populations.

## Introduction

Breast cancer is considered the most common type of cancer in females globally. However, breast cancer prognosis has significantly improved over the past four decades with ∼90% of patients now surviving ≥5 years. This has been attributed to advances in breast cancer screening, earlier detection, and treatment. Surgery, including breast-conserving surgery (BCS) and post-mastectomy breast reconstruction, and radiotherapy are the two most commonly used treatments for breast cancer. At the same time, both treatment modalities may cause side-effects that can adversely impact patients’ quality of life (QoL).^1^

Prediction models, including those based on machine learning (ML) techniques, are nowadays considered valuable tools in health care, offering the potential to forecast patients’ outcomes and tailor precision treatment plans accordingly.^2^ In the context of breast cancer surgery and radiotherapy, prediction models can help identify individuals or cohorts of patients who are at increased risk of adverse effects or unfavourable aesthetic outcomes. By utilising individual patient- and treatment-related data, prediction models can enhance informed decision making, leading to optimised treatment outcomes and improved patient QoL.^3^, ^4^, ^5^, ^6^

Nevertheless, the development and validation of reliable clinical prediction models poses several challenges, such as the heterogeneity of patient populations, variations in treatment approaches and protocols, and differences in scaling or classifying adverse outcomes. Therefore, the integration of diverse datasets and application of appropriate methodology are essential to capturing the complex interactions between various predictive variables.^7^ Anecdotally, researchers have used a variety of modelling approaches, from traditional statistical approaches such as logistic regression (LR) models to ML algorithms like random forests (RF), support vector machines (SVM), and gradient boosting regression, to investigate a range of predictors for outcomes such as capsular contracture, skin radiotoxicity, surgical site infection (SSI), and breast shape deformation.^8^, ^9^, ^10^, ^11^ Oleck et al. (2022)^12^ conducted a scoping review of predictive risk calculators for post-mastectomy reconstruction, which included 28 models with varying degrees of predictive performance and accuracy.

The aim of this paper was to extend previous work to systematically review the literature on prediction models for adverse outcomes in all breast cancer local treatment settings, i.e. surgery and radiotherapy, to synthesise the available evidence and evaluate the performance of existing models across the whole range of local treatment-related side-effects, in order to inform clinical practice and future research directions.

## Methodology

This review adhered to the Preferred Reporting Items for Systematic Reviews and Meta-Analyses (PRISMA) 2020 guidelines.^13^ It was based on the following questions: ‘what are the available prediction models for adverse outcomes following breast cancer surgery and radiotherapy’, ‘whether the existing models are accurate’, and ‘what is the clinical benefit’. The review protocol was registered (PROSPERO CRD420251017394).

### Inclusion and exclusion criteria

Studies were included if they reported on prediction models of breast appearance, outcome, complications, or toxicity following surgery or radiation treatment in women aged ≥18 years, assessing model performance including accuracy, calibration, and utility in clinical practice. Any study design, including observational studies or studies using clinical trial data, was included. Studies not reporting on prediction and those reporting breast assessment methods or scales only were excluded, as were conference abstracts, reviews, letters, or commentaries.

### Search strategy

A systematic literature search was carried out according to PRISMA guidelines in five electronic databases: PubMed, Medline (Ovid), Scopus, Web of Science, and CINAHL, using key words coupled with the relevant Medical Subject Heading terms, both of which were linked by either of the Boolean operators (Supplementary Table S1, available at https://doi.org/10.1016/j.esmorw.2026.100690), limited to English language publications and human subjects, but not limited by date of publication. Additional articles were identified through hand searches of the reference lists of included studies. The search covered studies published up to 15 June 2024.

### Data extraction

Abstracts of published studies were screened independently by two reviewers (HA, TR). Where there was disagreement between the reviewers, a third author was consulted (CJT). Data from eligible studies were extracted in a standardised format and quality-assessed according to the Transparent Reporting of a multivariable prediction model for Individual Prognosis Or Diagnosis + Artificial Intelligence (TRIPOD+AI) statement checklist^14^ (Supplementary Table S2, available at https://doi.org/10.1016/j.esmorw.2026.100690), which include items relevant to prediction model studies in terms of study design, sources of training and test/validation data, predictors, outcomes, data handling, model performance, limitations, and generalisability.

### Data synthesis and analysis

Due to the heterogeneity between studies, data synthesis was primarily qualitative, and a meta-analysis was not feasible. Included models are presented according to whether they predicted early or long-term adverse events in tabular format with summary statistics. Early adverse outcomes are defined as those that occur within the first 3 months (90 days) following treatments, and include wound healing issues, SSI, implant or flap failure, and acute radiotherapy skin reactions (erythema and desquamation). Long-term or late adverse outcomes may occur after 3 months up to many years following treatment, such as breast fibrosis (scarring), skin pigmentation changes and telangiectasia (dilated blood vessels under the skin), breast atrophy (volume loss), lymphoedema, and capsular contracture.

## Results

### Study selection

**Figure 1:**
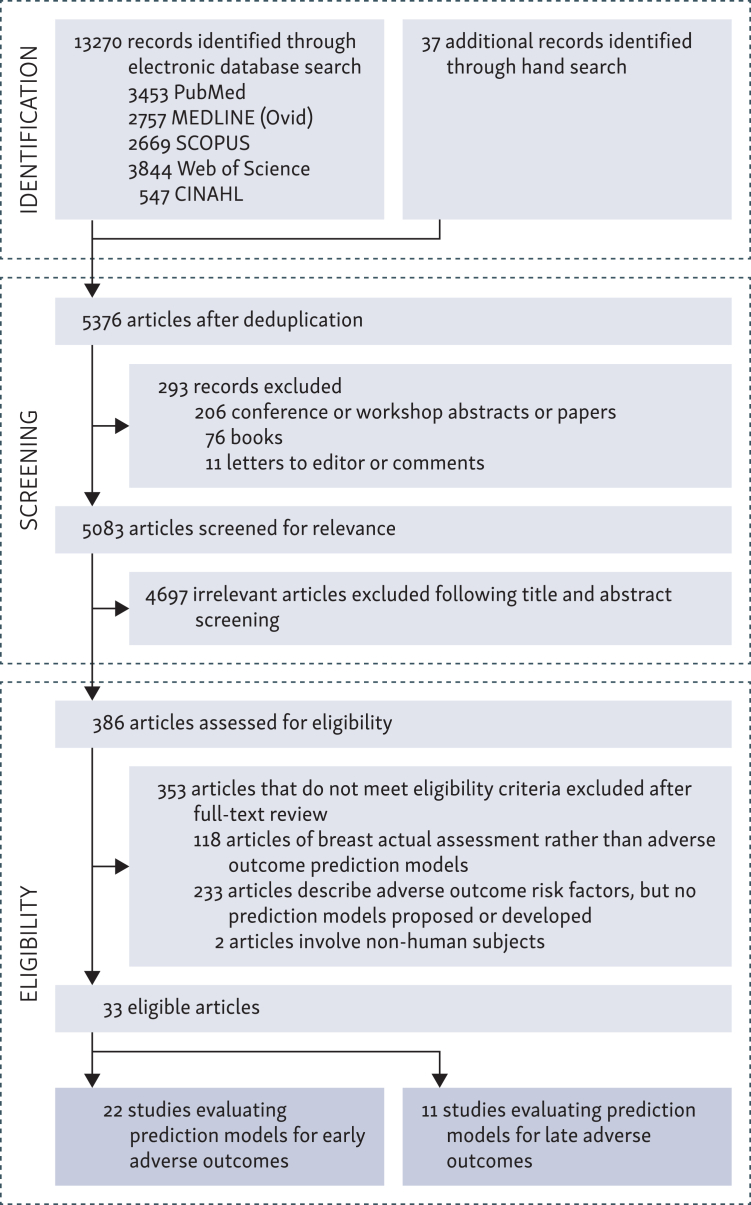
Preferred Reporting Items for Systematic Reviews and Meta-Analyses (PRISMA) flowchart showing the process of systematic literature record identification, screening, and eligibility.

The database and hand searches identified 13 307 records. Following removal of duplicates, a total of 5376 records proceeded to abstract screening. Before screening, 294 records were excluded as they were conference abstracts, reviews, or commentaries, and 4682 studies were excluded after screening their title and abstract, leaving 386 studies which were assessed for eligibility in full text. After excluding 118 studies that described breast assessment models or scales rather than prediction models, 233 studies that described risk factors and outcomes but lacked any model development, and 2 studies that involved non-human subjects, 33 studies were included in the final data synthesis (Figure 1).

### Study characteristics and results of individual studies

Individual studies that fulfilled the inclusion criteria were divided into two main categories: prediction models for (i) early and (ii) long-term adverse outcomes. Their main characteristics and results, including predictive performance, are described in the following sections:

### Prediction models for early adverse outcomes

#### Radiation-induced early skin toxicity

Several studies described prediction models of breast skin toxicity following radiotherapy. Rattay et al. (2020)^6^ successfully developed a prediction model for skin erythema [Common Terminology Criteria for Adverse Events (CTCAE) grade ≥2] in three combined observational datasets and externally validated the model using data from the multicentre REQUITE cohort study,^15^ demonstrating that their model could predict this type of acute skin radiation toxicity with an area under the curve (AUC) of 0.65, though with moderate calibration (Brier score 0.17). However, the study encountered challenges when attempting to validate a prediction model for the related endpoint desquamation, defined as CTCAE grade ≥3 radiation dermatitis/moist desquamation or CTCAE grade ≥1 skin ulceration according to the CTCAE (2017).^16^ Despite using the same REQUITE data, their model for desquamation failed to achieve satisfactory validation, indicating that predicting desquamation may require a different modelling approach.

Aldraimli et al. (2022)^17^ leveraged the same REQUITE cohort to focus on developing a new ML model to predict acute desquamation. They employed various ML algorithms, with the cost-sensitive RF model emerging as the most effective, achieving a sensitivity of 0.77, a specificity of 0.66, and an AUC of 0.77. This model underwent internal validation but still requires external validation to confirm its robustness across different patient populations (Table 1). Feng et al. (2022)^18^ developed a radiomics-based ML model for predicting radiation-induced acute skin toxicity (CTCAE grade ≥2) in breast cancer patients. This study follows the trajectory set by Rattay et al. (2020)^6^ and Aldraimli et al. (2022)^17^ in addressing the prediction of radiation-induced skin toxicity with more advanced methodologies. While Rattay et al. (2020)^6^ successfully validated a model for erythema but struggled with desquamation prediction, and Aldraimli et al. (2022)^17^ developed a ML model focusing on desquamation, Feng et al. (2022)^18^ further advanced the field by integrating radiomics features extracted from planning computed tomography images with clinical and dosimetric data. Their model, using a gradient boosting decision tree algorithm, achieved an excellent AUC of 0.998 in the training set and 0.911 in the validation set (Table 1).

**Table 1:** Summary of the studies evaluating predictive models for radiation-induced breast skin early toxicity

Study	No. of patients	Age	Intervention	Follow-up time	Predicted outcome	Selected predictors	Data source	Type of model developed	Model specification/algorithms used	Model validation	Model performance/metrics
Rattay et al. (2020)^6^	2031	Median age 58 years	BCS + EBRT ± bed boost	≤90 days	Breast radiation-induced dermatitis (erythema)	Patient, clinical, and treatment factors	Real world (REQUITE database)	Prediction model for acute erythema	Multivariate analysis	Bootstrapping (100) for internal validation. Externally validated.	AUC: 0.65, Brier score: 0.17
Aldraimli et al. (2002)^17^	2058	Median age 58 years	BCS + EBRT ± bed boost	≤90 days	Acute desquamation: CTCAE grade ≥3 radiation dermatitis (moist desquamation) or CTCAE grade ≥1 skin ulceration	Patient, clinical, and treatment factors	Real world (REQUITE database)	Acute desquamation risk score	8 ML models [naive Bayes (NB), logistic regression with ridge estimator, artificial neural networks with a multilayer perceptron architecture, support vector machine with polynomial kernel and logistic calibrator, K-nearest neighbour with K = 1, 3, 5, 7, 9, decision trees (C4.5), logistic model tree (LMT), and RF]	Internal validation using validation cohort—train–test split (50%–50%) and 10-fold cross-validation to reduce overfitting. No external validation.	AUC: 0.77, model sensitivity: 0.77, and specificity: 0.66
Feng et al. (2022)^18^	214	N/A	Breast cancer surgery + RT	N/A	RT-induced acute skin toxicity (grade ≥ 2)/radiodermatitis	(i) Laterality, (ii) quadrant position, (iii) histological type, (iv) T stage, (v) PR, (vi) hormone therapy, (vii) EQD2_all, (viii) Lotion application	Real world	Risk assessment model	Multivariate logistic regression gradient boosting decision tree (GBDT)	Cross-validation (five-fold) for internal validation (75% training and 25% validation). No external validation.	AUC for clinical and dosimetric features: 0.839 (training), 0.816 (validation) AUC for radiomic features: 0.998 (training), 0.911 (validation)
Cilla et al. (2023)^19^	129	N/A	Breast cancer surgery ± reconstruction + adjuvant WBR	≤6 months	RT-induced skin toxicity (RTOG ≥2)	(i) Spectrophotometric variables at time T0, (ii) BMI, (iii) PTV1, (iv) PTV2, (v) the dose fractionation scheme	Real world	Risk assessment model	3 ML models: SVM, CART, and LR	Cross-validation (five-fold) 25% training and 75% validation and Akaike information criterion (AIC) for internal validation. No external validation.	AUC: 0.664-0.816 SVM has best performance F-score of 88.7%-98.6%

AUC, area under the curve; BMI, body mass index; CART, classification and regression tree; CTCAE, Common Terminology Criteria for Adverse Events; EBRT, external beam radiotherapy; LR, logistic regression; ML, machine learning; N/A, not applicable; PR, progesterone receptor; PTV, planning target volume; RF, random forest; RT, radiotherapy; RTOG, Radiation Therapy Oncology Group; SVM, support vector machine; TE, tissue expander; WBR, whole breast radiotherapy.

Cilla et al. (2023)^19^ developed another predictive model for acute skin toxicity (grade ≥ 2) in breast cancer patients undergoing radiotherapy. In this study, quantitative spectrophotometric markers—melanin and erythema indices—were integrated with clinical variables to predict radiation-induced skin toxicity. Several statistical and ML models were developed, including LR, SVM, and classification and regression tree analysis, with the SVM model using the radial basis function kernel showing the best performance. This model achieved an accuracy of 89.8%, a precision of 88.7%, a recall of 98.6%, and an F-score of 93.3%. The study showed the potential of using spectrophotometry as a non-invasive tool for predicting skin toxicity and may offer a practical and interpretable alternative to other more complex methods and radiomics (Table 1). However, it is important to note that none of these models apart from Rattay et al. (2020)^6^ have been externally validated either in a temporally or a spatially segregated dataset, and there are as yet no models for early radiotherapy skin toxicity incorporating germline genomic data.

*Breast reconstruction complications.* Kim et al. (2014; 2015)^20^^,^^21^ and Khavanin et al. (2017)^22^ described the development and validation of the Breast Reconstruction Risk Assessment (BRA) score, a model designed to predict the likelihood of complications such as SSI, seroma, flap failure, or explantation (implant loss) following autologous and implant-based immediate breast reconstruction (IBR). Kim et al. (2014)^20^ initially developed and internally validated the BRA score using a large dataset from the American College of Surgery (ACS) National Surgical Quality Improvement Program (NSQIP). This work was further extended in Kim et al. (2015),^21^ where additional outcomes from the Tracking Operations and Outcomes for Plastic Surgeons (TOPS) database were integrated. Khavanin et al. (2017)^22^ conducted an external validation of the BRA score in a separate cohort, comprising two-stage IBR only, showing good calibration for SSI and seroma prediction, but not so much for implant loss, with moderate to good performance in terms of AUC, ranging from 0.69 to 0.78 depending on the outcome (Table 2).

**Table 2:** Summary of BRA-related studies evaluating predictive models for post-operative complications in breast reconstructive surgery

Study	No. of patients	Age	Intervention	Follow-up time	Predicted outcome	Selected predictors	Data source	Type of model developed	Model specification/algorithms used	Model validation	Model performance/metrics
Kim et al. (2014)^20^	16 069	N/A	IBR (implant and autologous)	≤30 days	Individualised SSI	Age, weight, height, ASA class, BMI, smoking, radiation, chemotherapy, hypertension, DM, clotting disorder, anticoagulation, CAD, PAD, dyspnoea, bilaterality of reconstruction	Real-world NSQIP database	Application of the BRA risk calculator for SSI	Multiple logistic regression model	Bootstrapping (1000 samples) for internal validation. Externally validated in different cohorts.	HL test *P* - 0.371 Brier score: 0.0357 C-statistic: 0.682
Kim et al. (2015)^21^	4439	N/A	IBR (implant and autologous)	≤30 days	Seroma, dehiscence, SSI, explantation, flap failure, reoperation, and overall complications	Age, BMI, current smoker, smoking, DM, ASA > 2	Real-world TOPS database	Extension of BRA model to include plastic surgery outcomes	Multiple logistic regression model	Bootstrapping (1000 samples) for internal validation. No external validation.	Corrected C-statistics 0.603-0.677 (0.699 uncorrected) HL test *P* = 0.167-0.609 Brier score: 0.007-0.063
Khavanin et al. (2017)^22^	855 (1333 breasts)	N/A	Two-stage IBR (TE/implant)	≤30 days	SSI and seroma	Age, weight, height, ASA class, BMI, smoking, radiation, chemotherapy, hypertension, DM, clotting disorder, anticoagulation, CAD, PAD, dyspnoea, bilaterality of reconstruction	Real-time NSQIP and TOPS datasets	Evaluation of the BRA for SSI and seroma	Multiple logistic regression model	External validation study of the BRA score model in two-stage IBR (TE/implant).	HL test *P* = 0.16-0.33 Brier score: 0.95-2.25%
Blough et al. (2018)^23^	903 (1365 breasts)	N/A	Two-stage IBR (TE/implant)	≤1 year	SSI, seroma, dehiscence/implant exposure, explantation	Age, weight, height, ASA class, BMI, smoking, radiation, chemotherapy, hypertension, DM, clotting disorder, anticoagulation, CAD, PAD, dyspnoea, bilaterality of reconstruction	Real world	BRA score enhanced beyond 30 days up to 1 year (BRA XL)	Five multiple logistic regression models (one for each complication plus one for overall complication)	Internal validation using key statistics. No external validation.	C-statistics: 0.674-0.739 HL tests: uniformly non-significant Brier scores: 0.027-0.154
Hansen et al. (2018)^24^	903 (1365 breasts)	N/A	Two-stage IBR (TE/implant)	≤1 year	Mastectomy skin flap necrosis (MSFN), SSI, seroma, dehiscence/implant exposure, explantation	Age, weight, height, ASA class, BMI, smoking, radiation, chemotherapy, hypertension, DM, clotting disorder, anticoagulation, CAD, PAD, dyspnoea, bilaterality of reconstruction	Real world	BRA score enhanced beyond 30 days up to 1 year (BRA XL)	Five multiple logistic regression models (one for each outcome)	Internal validation using key statistics. No external validation.	C-statistics: 0.674-0.739 HL tests: uniformly non-significant Brier scores: 0.027-0.154
O’Neill et al. (2019)^25^	415	N/A	Microvascular IBR	≤30 days	Surgical complications, medical complications, reoperation, and total or partial flap failure	Age, weight, height, ASA class, BMI, smoking, radiation, chemotherapy, hypertension, DM, clotting disorder, anticoagulation, CAD, PAD, dyspnoea, bilaterality of reconstruction	Real world	Evaluation of BRA score for microvascular IBR	Multiple logistic regression model	External validation of BRA in microvascular IBR.	C-statistics: 0.49-0.59 Brier scores: 0.09-0.44
O’Neill et al. (2020)^26^	1012	N/A	IBR + DBR (DIEP flap reconstruction)	N/A	Flap failure	Patient (age, BMI, comorbidities, smoking history), treatment (timing and laterality of reconstruction, history of radiation) factors	Real world	Risk assessment model	Machine learning resampling and decision tree classification models	Internal validation using validation cohort—train–test split (60%–40%). No external validation.	AUC: 0.67
Roy et al. (2019)^27^	351	N/A	IBR (DIEP flap)	≤90 days	Perioperative complications (microsurgical, surgical, and medical)	(i) BMI, (ii) prior radiotherapy, (iii) active smoking, (iv) comorbidity, (v) bilateral reconstruction, (vi) prior chemotherapy, (vii) age ≥ 65 years, (viii) prior hormonal therapy	Real world	Risk assessment model	Multivariable logistic regression	Internal validation using validation cohort of 100 patients—train–test split (71.5%–28.5%). No external validation.	C-statistic: 0.6 HL test *P* > 0.05
Martin et al. (2020)^28^	247	Average age 49.2 years	IBR (pre-pectoral expander)	≤30 days	SSI requiring i.v. abx or admission, seroma requiring drainage, dehiscence, explantation, skin necrosis, other expander-related complications	Age, BMI, current smoker, smoking, DM, ASA > 2	Real world	Application of BRA model in pre-pectoral expander IBR patients	As described in the original development of BRA model	External validation in patients undergoing pre-pectoral expander IBR.	BRA has poor predictive power in pre-pectoral breast reconstruction

Abx, antibiotics; ASA, American Society of Anesthesiologists; BMI, body mass index; BRA, Breast Reconstruction Risk Assessment; CAD, coronary artery disease; DBR, delayed breast reconstruction; DIEP, deep inferior epigastric perforator; DM, diabetes mellitus; IBR, immediate breast reconstruction; i.v., intravenous; IBR, immediate breast reconstruction; N/A, not applicable; NSQIP, National Surgical Quality Improvement Program; PAD, peripheral artery disease; SSI, surgical site infection; TE, tissue expander; TOPS, Tracking Operations and Outcomes for Plastic Surgeons.

Blough et al. (2018)^23^ and Hansen et al. (2018)^24^ validated and modified the BRA score, which was initially developed to predict 30-day complications from breast reconstruction surgery, to predict 1-year complications after implant-based breast reconstruction, including SSI, implant loss, and seroma formation (BRA score XL). Using their own institutional database, they observed that less than a third of all complications occurred in the initial 30-day window following surgery. Reported AUCs for these extended models ranged from 0.661 to 0.739 and Brier scores from 0.027 to 0.154 (Table 2).

O’Neill et al. (2019)^25^ tested the ability of the BRA score to predict complications from microvascular breast reconstruction, specifically focusing on the deep inferior epigastric perforator (DIEP) flap procedures. They found that while the BRA score provided had some clinical utility, it underperformed in predicting medical complications, flap failure, and donor-site morbidity, complications which are specific to microvascular free-flap surgery with its longer operating time. AUCs for four models ranged from 0.49 to 0.59 with Brier scores from 0.09 to 0.44, indicating a lack of validation for microvascular reconstruction. This motivated the authors to develop a new model based on an ensemble ML decision tree for medical and surgical complications following DIEP flap reconstruction albeit with a moderate average AUC of 0.67 in their internal validation (test) cohort (O’Neill et al., 2020).^26^ In the same surgical setting of microvascular reconstruction, Roy et al. (2019)^27^ developed and validated a categorical prediction model using data from the same institution, stratifying patients into low-, intermediate-, and high-risk groups (Table 2), with an AUC of 0.70.

Martin et al. (2020)^28^ attempted to validate the 30-day BRA score for pre-pectoral implant-based reconstruction, a technique gaining popularity due to its less invasive nature compared with submuscular implant placement. Notably, half of the 30-day complications observed in their validation cohort were due to skin necrosis, a complication not included in the original BRA score model. For occurrence of any of the BRA score-predicted complications, the model remained well calibrated though discriminated poor with AUC <0.60. Further modification and external validation of the BRA score for these surgical settings are awaited (Table 2).

#### ACS NSQIP—Surgical Risk Calculator

Several publications have described the development and validation of the Surgical Risk Calculator (SRC), an LR model based on the ACS NSQIP dataset. Fischer et al. (2013)^29^ developed and internally validated a categorical prediction model for complications following autologous and implant-based IBR, classifying patients into four risk groups (low, intermediate, high, very high), with good predictive performance. However, their model only predicts the risk of any complication rather than distinct complications (Table 3). It has not yet been externally validated.

**Table 3:** Summary of ACS NSQIP-related studies evaluating predictive models for post-operative complications in breast surgery

Study	No. of patients	Age	Intervention	Follow-up time	Predicted outcome	Selected predictors	Data source	Type of model developed	Model specification/algorithms used	Model validation	Model performance/metrics
Fischer et al. (2013)^29^	12 129	N/A	IBR (implant and autologous)	≤30 days	Composite post-operative complication	(i) Obesity, (ii) autologous reconstruction, (iii) active smoking, (iv) ASA	Real-world NSQIP database	Risk assessment scale (IBRRAS) (four risk groups: low, intermediate, high, very high)	Multivariate logistic regression for IBR composite risk score model development by assigning rounded odds ratios to each variable and summing risk factors for each patient	Internal validation using validation cohort—train–test split (2 : 1). Externally validated in different cohorts.	No significant difference between the model cohort and validation cohort (*P* > 0.05)
O’Neil et al. (2016)^30^	515 (759 breast)	N/A	Abdominal autologous breast reconstruction	≤30 days	Any complications, serious complications	Age, functional status, emergency case, ASA, wound class, steroid use, ascites, sepsis, use of ventilator, disseminated cancer, diabetes, hypertension, previous cardiac event, CHF, dyspnoea, smoker, COPD, dialysis, AKI, BMI	Real-world NSQIP database	Evaluation of ACS SRC in breast free-flap reconstruction	Bivariate analysis was carried out to compare overall rate of predicted risk of complications with the observed risk of complications	External validation of ACS SRC is attempted in patients with autologous breast reconstruction.	Hosmer–Lemeshow test was non-significant AUC or C-statistics: 0.548 Brier score was higher than that reported in the original ACS calculator development (0.094 versus 0.069)
Gonzalez-Woge et al. (2021)^31^	385	N/A	Different breast cancer surgeries	≤30 days	Any complications, serious complications	Age, functional status, emergency case, ASA, steroid use, sepsis, use of ventilator, disseminated cancer, diabetes, hypertension, heart failure, dyspnoea, smoker, COPD, dialysis, AKI, BMI	Real-time INCan database	Evaluation of ACS SRC for breast cancer surgery in a Mexican cohort	Multivariate logistic regression	External validation of ACS SRC is attempted in Mexican patients.	AUC: 0.617 for any complication, 0.682 for serious complications Hosmer–Lemeshow test significant (<0.05) for both outcomes Brier scores were 0.102 for any complication and 0.048 for serious complication
Dube et al. (2022)^10^	210	N/A	Breast cancer (primary or recurrent) surgery ± reconstruction	≤30 days	SSI, serious post-op complication	Age, functional status, emergency case, ASA, steroid use, sepsis, use of ventilator, disseminated cancer, diabetes, hypertension, heart failure, dyspnoea, smoker, COPD, dialysis, AKI, BMI	Real-world NSQIP database	Evaluation of ACS SRC for breast cancer surgery in English cohort	Univariate logistic regression models	External validation of ACS SRC is attempted in English patients.	SSI and serious complications prediction: moderate accuracy SSI, AUC of 79.4%, sensitivity of 63.6%, and specificity of 91.7%
Jonczyk et al. (2021)^32^	163 613	N/A	1 of 5 procedures (partial mastectomy, total mastectomy, implant/TE reconstruction, or free-flap reconstruction)	≤30 days	Acute post-operative complications (infectious, hematologic, internal organ, and overall complications)	Age, race, ethnicity, BMI, smoking status, glucocorticoid or anticoagulation use, unintentional weight loss, DM, hypertension, dyspnoea, COPD, CHF, diagnosis, stage 4 metastatic cancer, surgeon specialty, type of anaesthesia, axillary lymph node management, preoperative functional status, anaesthesia type, transfer status, admission status, and admission quarter	Real-world NSQIP database	The Breast Cancer Surgery Risk Calculator (BCSRC)	Four multivariate logistic regression models (one for each endpoint) for risk calculator model development	Bootstrap resampling (300 times) used for internal validation. Externally validated by Jonczyk et al. (2023).^33^	AUC: overall, 0.70, infectious 0.67, hematologic 0.84, and internal organ 0.74 Accuracy (Brier scores): overall 0.05-0.04, infectious 0.04-0.03, internal organ 0.006-0.003, and hematologic 0.012-0.009 Model calibration using the Hosmer–Lemeshow statistic found all *P* > 0.05
Jonczyk et al. (2023)^33^	60 144	N/A	1 of 5 procedures (partial mastectomy, total mastectomy, implant/TE reconstruction, or free-flap reconstruction)	≤30 days	Acute post-operative complications (infectious, hematologic, internal organ, and overall complications)	Age, race, ethnicity, BMI, smoking status, glucocorticoid or anticoagulation use, unintentional weight loss, DM, hypertension, dyspnoea, COPD, CHF, diagnosis, stage 4 metastatic cancer, surgeon specialty, type of anaesthesia, axillary lymph node management, preoperative functional status, anaesthesia type, transfer status, admission status, and admission quarter	Real-world NSQIP database	The Breast Cancer Surgery Risk Calculator (BCSRC)	Four multivariate logistic regression models (one for each endpoint) for risk calculator model development	External validation for Breast Cancer Surgery Risk Calculator (BCSRC) for post-operative complications.	AUC during external validation for each model was ∼0.70 Accuracy or Brier scores were all between 0.04 and 0.003 Model calibration using the Hosmer–Lemeshow statistic found all *P* > 0.05

ACS SRC, American College of Surgeons Surgical Risk Calculator; AKI, acute kidney injury; ASA, American Society of Anesthesiologists; AUC, area under the curve; BMI, body mass index; CHF, congestive heart failure; COPD, chronic obstructive pulmonary disease; IBR, immediate breast reconstruction; N/A, not applicable; NSQIP, National Surgical Quality Improvement Program; SSI, surgical site infection.

External validation of the ACS NSQIP SRC was attempted in unselected Mexican and English breast surgical patient cohorts. Gonzalez-Woge et al. (2021)^31^ and Dube et al. (2022)^10^ found that SRC under-predicted complications with moderate discrimination (AUC 0.617 for any complication and 0.682 for serious complications), whereas the results were more promising in Dube et al. (2022)^10^ with an AUC of 0.794 for SSI and 0.845 for serious complications, respectively (Table 3). O’Neill et al. (2016)^30^ focused their investigation on validating the tool for microvascular reconstruction with relatively poor performance (AUC 0.548). The authors also noted an absence of complications specific to free-flap reconstruction, such as flap failure, from the SRC model (Table 3).

In two studies by Jonczyk et al. (2021; 2023)^32^^,^^33^ using a larger NSQIP dataset of patients treated between 2005 and 2018, the SRC was re-trained and re-calibrated for patients undergoing either BCS or mastectomy to predict four composite outcomes: overall, infectious, hematologic, and internal organ complications, and validated in a more recent (2018-2020) NSQIP dataset with an overall moderate average AUC of 0.70 and Brier scores between 0.04 and 0.003 depending on predicted outcome. The validated and updated models are available on the Breast Cancer Surgery Risk Calculator (BCSRC) platform (www.breastcalc.org) (Table 3).

*Other models*. Nelson et al. (2015)^34^ developed a categorical model in a single-institutional dataset of patients undergoing autologous breast reconstruction with three risk groups, showing that high-risk patients have an 86% risk of wound healing complications, compared with a 33% risk in patients with few risk factors, although data on model performance were not presented. Park et al. (2020)^35^ developed a model and risk score for overall complications in two-stage IBR with an AUC of 0.732 and 0.731, respectively. Frey et al. (2020)^36^ developed a model for overall complications in nipple-sparing mastectomy in a single-institution dataset with an AUC of 0.668 in their split internal validation cohort. All these models included smoking and body mass index (BMI) (obesity) among the predictors (Table 4).

**Table 4:** Summary of studies evaluating prediction models for delayed wound healing following breast surgery

Study	No. of patients	Age	Intervention	Follow-up time	Predicted outcome	Selected predictors	Data source	Type of model developed	Model specification/algorithms used	Model validation	Model performance/metrics
Nelson et al. (2015)^34^	682 (1033 breast)	N/A	Free autologous reconstruction	3 weeks	Breast and donor site delayed wound healing (wounds requiring dressing changes for > 3 weeks)	Class I-III obesity, current and past smoking, bilateral reconstruction, and receipt of any vasopressor during reconstruction	Real world	Risk assessment model (three risk groups: low, intermediate, and high)	Multivariate logistic regression	Backward stepwise bootstrap regression (1000 random samples) for internal validation. No external validation attempted	N/A
Park et al. (2020)^35^	619 (653 breast)	N/A	Two-stage IBR (TE/implant)	6 months	One or more (seroma, hematoma, infection, mastectomy flap necrosis ‘required debridement’, delayed wound healing, reconstruction failure, revision surgery)	Smoking history, radiotherapy, and a final inflation volume of ≥450 ml	Real world	Risk assessment model	Multivariate analysis—stepwise logistic regression	Internal validation using key statistics. No external validation attempted	AUC: 0.732 and 0.731 for the logistic regression model and risk-scoring system, respectively (*P* = 0.975). *P* values non-significant
Frey et al. (2020)^36^	1070	N/A	IBR with nipple-sparing mastectomy	N/A	Overall complications (including delayed wound healing)	Age, active smoking, DM, BMI, therapeutic mastectomy, prior chemotherapy, prior radiation, adjuvant radiation, adjuvant chemotherapy, mastectomy weight, mastectomy incision, reconstruction type	Real world	Risk assessment model	Multivariate logistic regression	Internal validation using validation cohort train–test split (50.2%-49.8%). No external validation	AUC: 0.668

AUC, area under the curve; BMI, body mass index; DM, diabetes mellitus; IBR, immediate breast reconstruction; N/A, not applicable; TE, tissue expander.

### Prediction models for long-term adverse outcomes

#### Radiation-induced late toxicity

Mbah et al. (2018)^37^ developed a prediction model for the long-term radiation toxicity endpoints oedema, fibrosis, retraction, and pigmentation in breast cancer patients undergoing radiotherapy. They modelled overall patient radiosensitivity and multiple individual toxicity endpoints simultaneously using LR based on maximum likelihood estimators (MLEs). MLEs of a given predictive variable were further improved by combining other MLEs for the same variable for different toxicity endpoints, called James–Stein estimator (JSE), resulting in a lower mean squared error. Based on the JSE, 19 variables were included in their prediction model, including breast volume, chemotherapy, age, nodal irradiation, and candidate genetic variants. This study included data and genotypes from 269 patients and is yet to be externally validated (Table 5). Hammer et al. (2017)^4^ developed a dosimetric prediction model for CTCAE grade ≥2 radiation-induced subcutaneous fibrosis in the boost area in patients undergoing three-dimensional conformal radiotherapy with a simultaneous integrated boost technique for early-stage breast cancer, with patient age, the volume of the breast receiving >55 Gy (V55), and the maximum radiation dose (Dmax) as predictors. This model demonstrated moderate predictive performance with an AUC of 0.66. However, it still requires external validation, including for patients treated with other fractionation schedules (Table 5).

**Table 5:** Summary of studies evaluating prediction models for breast long-term radiotoxicity and adverse cosmetic outcomes

Study	No. of patients	Age	Intervention	Follow-up time	Predicted outcome	Selected predictors	Data source	Type of model developed	Model specification/algorithms used	Model validation	Model performance/metrics
Mbah et al. (2018)^37^	269	N/A	BCS + WBI	2 years	Late radiotoxicity (five endpoints: oedema, retraction, fibrosis, pigmentation, BCCT.core)	Breast volume, chemotherapy, older age, and SATB2 rs2881208 SNP	Real world	Risk assessment model	MLE- and JSE-based models	100 rounds of a five-fold cross-validation for internal validation. No external validation.	Accuracy: JSE: 66% correct classification. MLE: 55% correct classification
Hammer et al. (2017)^4^	546	Median age 65 years	BCS + 3D-CRT-SIB	5 years	Grade ≥2 radiation-induced fibrosis in the boost area	Patient age, breast volume receiving P55 Gy (V55 CTV breast), and the maximum radiation dose in the breast (Dmax)	Real world	Risk assessment model	Multivariate logistic regression	Internal validation using bootstrapping and sequential forward variable selection. No external validation.	AUC: 0.66 HL test non-significant *P* = 0.42
Vos et al. (2015)^3^	67 (69 breast)	N/A	BCS + RT	33 months (median)	Cosmetic outcome assessed by panel, BRA, patient	Tumour/breast volumes ratio, tumour location, specimen weight	Real world	Breast cosmesis prediction tool	Multivariate linear regression	Internal validation using key statistics, No external validation.	AUC: 0.83
Manie et al. (2018)^5^	64	Median age 47 years	Mastectomy + extended latissimus dorsi flap IBR	N/A	Cosmetic outcome assessed by panel and patient	N/A	Real world	Breast cosmesis prediction tool	N/A	No internal or external validation.	N/A
Kindts et al. (2019)^38^	121	Median age 60 years	BCS + WBI followed by boost to the tumour bed	1 year	Late unfavourable aesthetic outcome—late radiotoxicity	Clinicopathological factors (seroma and axillary lymphadenectomy) and radiation dose-volume metrics (V55)	Real world	Risk assessment (NTCP) model	Multivariable logistic regression	Bootstrapping (10 000) for internal validation. No external validation.	AUC 0.75 HL test *P* value: non-significant
Meshulam-Derazon et al. (2024)^39^	136	Average age 49.3 years	BCS + RT	1 year	Poor cosmetic/shape outcome	BMI, removed tissue volume, tumour location	Real world	Risk assessment model	Logistic regression	No internal or external validation.	N/A
Naoum et al. (2022)^40^	1617	N/A	Breast reconstruction: autologous, TE/implant, direct-to-implant ± RT	6.6 years (median)	(i) Infection/necrosis requiring debridement, (ii) capsular contracture requiring capsulotomy, (iii) absolute and (iv) overall implant failure	Smoking, DM, BMI, radiotherapy, total LNs sampled, total malignant LNs, reconstruction time, incision type, chemotherapy, mesh type, ethnicity, menopause status	Real world (Research Electronic Data Capture database)	Risk assessment nomograms	Four multivariate logistic regression models used (one for each endpoint)	Cross-validation (10-fold) for internal validation. No external validation.	AUC: 68%-76%
Bavaro et al. (2023)^8^	59	Median age 47 years	IBR (implant) + 3D CRT	≥18 months	Capsular contracture	PgR, ER, lymph node status, histological grading, histological subtype, Ki67 expression, and molecular subtype	Real world	Risk assessment model	Classification algorithms: RF, XGBoost, SVM	10 rounds of a 10-fold cross-validation for internal validation. No external validation.	XGBoost, SVM, RF (respectively): AUC: 68%, 66%, 65%. Accuracy: 68%, 66%, 64%. Sensitivity: 64%, 64%, 82%. Specificity: 74%, 65%, 48%

3D-CRT-SIB, three-dimensional conformal radiotherapy with a simultaneous integrated boost; BCS, breast-conserving surgery; BMI, body mass index; CTV, clinical target volume; DM, diabetes mellitus; ER, estrogen receptor; HL, Hosmer-Lemeshow; IBR, immediate breast reconstruction; JSE, James–Stein estimator; LN, lymph node; MLE, maximum likelihood estimator; N/A, not applicable; PgR, progesterone receptor; RF, random forest; RT, radiotherapy; SVM, support vector machine; WBI, whole breast irradiation.

#### Adverse breast cosmesis

Several prediction models have been developed for adverse breast cosmesis following BCS and radiotherapy. Vos et al. (2015)^3^ investigated the effect of tumour volume to breast volume, tumour location, and specimen weight on cosmetic outcome by panel assessment in 69 patients. Their model showed moderate performance with a C-index of 0.64. Kindts et al. (2019)^38^ developed and attempted to validate a model for unfavourable cosmetic outcome scored by BCCT.core software^41^ with the variables seroma, axillary lymph node dissection (ALND), and V55. AUC was 0.75 in the development cohort treated between 2009 and 2014 and 0.66 in the validation cohort of patients who were treated at the same centre in 2015-2016. The authors then modified their published model by including V45 and retaining seroma but not ALND, with the new model achieving an AUC of 0.75 in the validation cohort. Meshulam-Derazon et al. (2024)^39^ developed models for adverse cosmetic outcome and adverse breast shape after BCS as determined by panel assessment like in Vos et al. (2015)^3^ to identify patients who might benefit from oncoplastic intervention. The final published models incorporated BMI and various chest wall and breast measurements including tumour position within the breast, although they did not provide performance metrics and the models have not been externally validated (Table 5).

For adverse cosmesis following post-mastectomy breast reconstruction, Manie et al. (2018)^5^ developed a model for patients undergoing immediate reconstruction with an extended latissimus dorsi using BMI and breast cup size. They provided a simple nomogram and showed that BMI >33 kg/m^2^ was predictive of unfavourable cosmetic outcome regardless of breast cup size. Their study had a relatively small sample size of 64 patients and external validation has not been carried out (Table 5). Naoum et al. (2022)^40^ and Bavaro et al. (2023)^8^ developed prediction models for capsular contracture following implant-based breast reconstruction and radiotherapy. Naoum et al. (2022)^40^ used LASSO penalized regression to select predictors in a large dataset of 1619 patients who underwent reconstruction between 1997 and 2007 and presented a nomogram, whereas Bavaro et al. (2023)^8^ applied ML classification models to a small patient dataset (*n* = 59) with the extreme gradient boosting classifier achieving the highest AUC of 0.68. Neither of these models have been externally validated.

#### Multiscale prediction models of breast appearance

Three publications described the development of a multiscale finite element model (FEM) to predict breast appearance following BCS. The aim of these studies was to leverage computational modelling and ML to simulate breast healing and deformation over time. Garbey et al. (2013)^42^ introduced a two-dimensional simulation framework that integrates mechanical tissue deformation and biological healing models. They utilised cellular automata to model the healing process at the cellular level and FEM to simulate tissue deformation under gravity and other mechanical forces. Magnetic resonance imaging (MRI) was used to provide initial breast geometry, although patient-specific mechanical properties were not fully captured (Table 6). Vavourakis et al. (2016)^43^ expanded the modelling framework to three-dimensional simulations incorporating FEM and continuum mechanics to simulate tissue deformation, coupled with biological healing models. Their model simulated the healing process over several months, accounting for factors such as tissue stiffness, inflammation, and remodelling. Their computational framework integrates clinical and imaging data (e.g. MRI) from breast cancer patients to create patient-specific models that predict breast shape and appearance over time (Table 6).

**Table 6:** Summary of studies evaluating multiscale prediction models of breast appearance following BCS

Study	No. of patients	Age	Intervention	Follow-up time	Predicted outcome	Selected predictors	Data source	Type of model developed	Model specification/algorithms used	Model validation	Model performance/metrics
Garbey et al. (2013)^42^	N/A	N/A	BCS	POD 1 onwards	Cosmesis outcome	MRI images, mechanical, biological, and molecular variables	Real world	2D multiscale biomechanical model/FEM of (proof of concept)	Multiscale ML modelling coupled with mechanical and biological models	No internal validation.	N/A
Vavourakis et al. (2016)^43^	4	N/A	BCS	Post-operative day 1 onwards up to 1 year	Breast tissue deformation and wound healing	MRI images, mechanical, biological, and molecular variables	Real world	3D multiscale biomechanical model/FEM	Multiscale ML modelling coupled with mechanical and biological models	Follow-up data, in the form of 3dMD surface scans, were acquired 6-12 months after surgery for each patient and compared directly with the predicted surgical outcome. No external validation.	High accuracy: The mean surface distances between the simulation and the follow-up optical surface scan range between 2.8 and 4.1 mm. This indicates an excellent simulation accuracy.
Zolfagharnasab et al. (2018)^11^	N/A	N/A	BCS simulation	1 year	Breast shape deformation	MRI images, mechanical, biological, and molecular variables	In-house semi-synthetic dataset	3D multiscale biomechanical model/FEM	Regression algorithms:	Leave-one-patient-out (LOPO) cross-validation technique. No external validation.	N/A

2D, two-dimensional; BCS, breast-conserving surgery; FEM, finite element model; GBR, gradient boosting regression; ML, machine learning; MOR, multi-output regression; MRI, magnetic resonance imaging; N/A, not applicable; RF, random forest.

Zolfagharnasab et al. (2018)^11^ focused on overcoming the time and resource demands of biomechanical modelling (FEM) by introducing ML techniques into the modelling process. The authors used SVM and artificial neural networks to generate predictions of cosmetic outcomes based on various features in a semi-synthetic dataset derived from in-house breast MRI images to predict complex outcomes such as breast contour and symmetry (Table 6).

## Discussion

The aim of this paper was to systematically review the literature on prediction models for adverse outcomes following breast cancer surgery and radiotherapy. It provides a qualitative synthesis of 33 studies which modelled a range of early and long-term adverse events, SSI, delayed wound healing, capsular contracture, radiation-induced skin toxicity, and breast deformity. The prediction tools ranged from traditional statistical models, such as LR, to biomechanical modelling and ML algorithms, which are increasingly being applied in clinical research. The most frequently reported models to date are the ACS NSQIP and the Breast Cancer Surgical Risk Calculators and the BRA score, all of which predict the risk of early complications following breast surgery and reconstruction. Despite the relatively large training datasets used in the development of these prediction models, they suffer from a relative lack of external validation, thus limiting their generalisability across different clinical settings and countries.

There is as yet no externally validated prediction model for long-term adverse outcomes following breast surgery or radiotherapy. The model developed by Kindts et al. (2019)^38^ failed to validate in a later cohort recruited at the same centre, although the authors modified and re-calibrated their clinico-dosimetric model and achieved moderate performance in the test cohort. Apart from Naoum et al. (2022),^40^ these studies used relatively modest training datasets with a maximum of a few hundred patients. Applied to predicting early radiation-induced skin toxicity as well as long-term capsular contracture and breast appearance, ML models have emerged with improved predictive performance compared with traditional statistical approaches. They also have the potential to incorporate a wider range and number of patient variables, such as imaging data and genomic markers,^44^ and may be applied to more complex endpoints. Published ML-based models have also been shown to outperform traditional statistical models by incorporating advanced data sampling techniques.^6^

The findings of this systematic review align with previous literature, which has identified the need for robust prediction models in breast cancer treatment to anticipate adverse outcomes and optimise patient care.^12^^,^^45^^,^^46^ While significant strides have been made in developing these models, the lack of external validation remains a challenge to clinical implementation. External validation is necessary for models to be used across diverse populations and settings,^47^ yet it was notably absent in most studies included in this review. Studies that included external validation, such as those evaluating the ACS NSQIP SRC and BRA score, revealed that many models require re-calibration for specific surgical techniques, such as microsurgical or pre-pectoral breast reconstruction. A sample size of ≥100, and ideally ≥200, is a crucial requirement for conducting external validation.^48^ Notably, while they frequently reported internal validation with split training–test datasets, none of the ML-based studies included in this review have extended validation to external cohorts, which may be due to lack of available multi-dimensional datasets.

For more complex endpoints concerning breast appearance, multiscale FEMs can account for the interaction between mechanical stress, gravity, and biological healing processes. However, these are computationally intensive, but in combination with ML used to extract key features from the models, they show promise in predicting post-operative appearance for use in the clinic. Of course, these emerging models will require external validation.

### Study limitations

Although this systematic review has several strengths by including both parametric statistical and ML models covering the whole range of adverse outcomes following breast surgery and radiotherapy, there are some limitations. The search process may have missed relevant studies because this systematic review was limited to the available published data. This limitation was lessened by thoroughly screening >300 full-text records to ensure that only accurate prediction models were involved. The preponderance of models that lack external validation restricts their utility in clinical practice. Without external validation, the models’ performance in different patient populations and clinical settings remains uncertain.^47^ Furthermore, the heterogeneity of patient populations, treatment modalities, and outcome measures across the included studies pose challenges for risk of bias estimation and quantitative evidence synthesis. Many published models were developed in relatively small patient cohorts, which may limit their generalisability and reliability. Finally, the majority of published models focused on short-term outcomes, with fewer models addressing long-term outcomes such as late radiation toxicity and breast appearance.

Future research should prioritise the external validation of existing models across diverse clinical settings and patient populations, with modification to improve their performance and calibration if required. This step is essential for ensuring that prediction models can be reliably applied in routine clinical practice. Additionally, given the high survival rates in early breast cancer, long-term outcomes pose a major concern as they can significantly impact QoL. Therefore, there is a pressing need to develop prediction models that account for long-term outcomes, such as breast appearance, shape, and shrinkage (atrophy), the latter being an adverse outcome of radiotherapy. Furthermore, exploration of ML techniques, particularly those that incorporate imaging and genomic data, could enhance the predictive power of models and allow for more personalised risk assessments and better-tailored treatment plans for breast cancer patients.

### Conclusion

This systematic review demonstrates that the majority of prediction models for adverse outcomes following breast cancer surgery and radiotherapy are not yet ready for widespread clinical implementation across diverse populations and clinical settings due to their lack of validation and immature technology development. It also highlights a relative lack of prediction models for long-term side-effects and more complex outcomes, such as cosmetic breast appearance and QoL, suggesting areas for future research.

## References

[1] Nardin S., Mora E., Varughese F.M. (2020). Breast cancer survivorship, quality of life, and late toxicities. Front Oncol.

[2] Rubinger L., Gazendam A., Ekhtiari S., Bhandari M. (2023). Machine learning and artificial intelligence in research and healthcare. Injury.

[3] Vos E.L., Koning A.H.J., Obdeijn I.M. (2015). Preoperative prediction of cosmetic results in breast conserving surgery. J Surg Oncol.

[4] Hammer C., Maduro J.H., Bantema-Joppe E.J. (2017). Radiation-induced fibrosis in the boost area after three-dimensional conformal radiotherapy with a simultaneous integrated boost technique for early-stage breast cancer: a multivariable prediction model. Radiother Oncol.

[5] Manie T., Farahat A., Hashem T. (2018). Preoperative estimation of cosmetic outcomes after immediate breast reconstruction with extended latissimus dorsi flap: a simple prediction model. JPRAS Open.

[6] Rattay T., Seibold P., Aguado-Barrera M.E. (2020). External validation of a predictive model for acute skin radiation toxicity in the REQUITE breast cohort. Front Oncol.

[7] Cardoso J.S., Silva W., Cardoso M.J. (2020). Evolution, current challenges, and future possibilities in the objective assessment of aesthetic outcome of breast cancer locoregional treatment. Breast.

[8] Bavaro D.A., Fanizzi A., Iacovelli S. (2023). A machine learning approach for predicting capsular contracture after postmastectomy radiotherapy in breast cancer patients. Healthcare (Basel).

[9] Chen M.F., Chen W.C., Lai C.H., Hung C.H., Liu K.C., Cheng Y.H. (2010). Predictive factors of radiation-induced skin toxicity in breast cancer patients. BMC Cancer.

[10] Dube M., Nour S., Shafee A.S., Lahart I., Carmichael A. (2022). A prospective evaluation of the American College of Surgeons Surgical Risk Calculator as a predictor of complications for breast surgery. Ann R Coll Surg Engl.

[11] Zolfagharnasab H., Bessa S., Oliveira S.P. (2018). A regression model for predicting shape deformation after breast conserving surgery. Sensors (Basel).

[12] Oleck N.C., Biswas S., Shammas R.L., Naga H.I., Phillips B.T. (2022). An ounce of prediction is worth a pound of cure: risk calculators in breast reconstruction. Plast Reconstr Surg Glob Open.

[13] Page M.J., Moher D., Bossuyt P.M. (2021). PRISMA 2020 explanation and elaboration: updated guidance and exemplars for reporting systematic reviews. Br Med J.

[14] Collins G.S., Moons K.G.M., Dhiman P. (2024). TRIPOD+AI statement: updated guidance for reporting clinical prediction models that use regression or machine learning methods. Br Med J.

[15] Seibold P., Webb A., Aguado-Barrera M.E. (2019). REQUITE: a prospective multicentre cohort study of patients undergoing radiotherapy for breast, lung or prostate cancer. Radiother Oncol.

[16] National Cancer Institute (2017). https://ctep.cancer.gov/protocoldevelopment/electronic_applications/ctc.htm.

[17] Aldraimli M., Osman S., Grishchuck D. (2022). Development and optimization of a machine-learning prediction model for acute desquamation after breast radiation therapy in the multicenter REQUITE cohort. Adv Radiat Oncol.

[18] Feng H., Wang H., Xu L. (2022). Prediction of radiation-induced acute skin toxicity in breast cancer patients using data encapsulation screening and dose-gradient-based multi-region radiomics technique: a multicenter study. Front Oncol.

[19] Cilla S., Romano C., Macchia G. (2023). Machine-learning prediction model for acute skin toxicity after breast radiation therapy using spectrophotometry. Front Oncol.

[20] Kim J.Y.S., Khavanin N., Jordan S.W. (2014). Individualized risk of surgical-site infection: an application of the breast reconstruction risk assessment score. Plast Reconstr Surg.

[21] Kim J.Y.S., Mlodinow A.S., Khavanin N. (2015). Individualized risk of surgical complications: an application of the breast reconstruction risk assessment score. Plast Reconstr Surg Glob Open.

[22] Khavanin N., Qiu C.S., Mlodinow A.S. (2017). External validation of the breast reconstruction risk assessment calculator. J Plast Reconstr Aesthet Surg.

[23] Blough J.T., Vu M.M., Qiu C.S. (2018). Beyond 30 days: a risk calculator for longer term outcomes of prosthetic breast reconstruction. Plast Reconstr Surg Glob Open.

[24] Hansen N., Espino S., Blough J.T., Vu M.M., Fine N.A., Kim J.Y.S. (2018). Evaluating mastectomy skin flap necrosis in the extended breast reconstruction risk assessment score for 1-year prediction of prosthetic reconstruction outcomes. J Am Coll Surg.

[25] O’Neill A.C., Murphy A.M., Sebastiampillai S., Zhong T., Hofer S.O.P. (2019). Predicting complications in immediate microvascular breast reconstruction: validity of the breast reconstruction assessment (BRA) surgical risk calculator. J Plast Reconstr Aesthet Surg.

[26] O’Neill A.C., Yang D., Roy M., Sebastiampillai S., Hofer S.O.P., Xu W. (2020). Development and evaluation of a machine learning prediction model for flap failure in microvascular breast reconstruction. Ann Surg Oncol.

[27] Roy M., Sebastiampillai S., Haykal S., Zhong T., Hofer S.O.P., O’Neill A.C. (2019). Development and validation of a risk stratification model for immediate microvascular breast reconstruction. J Surg Oncol.

[28] Martin S., Turner E., Nguyen A., Thornton B., Nazerali R.S. (2020). An evaluation of the utility of the breast reconstruction risk assessment score risk model in prepectoral tissue expander breast reconstruction. Ann Plast Surg.

[29] Fischer J.P., Wes A.M., Tuggle C.T., Serletti J.M., Wu L.C. (2013). Risk analysis and stratification of surgical morbidity after immediate breast reconstruction. J Am Coll Surg.

[30] O’Neill A.C., Bagher S., Barandun M., Hofer S.O.P., Zhong T. (2016). Can the American College of Surgeons NSQIP surgical risk calculator identify patients at risk of complications following microsurgical breast reconstruction?. J Plast Reconstr Aesthet Surg.

[31] Gonzalez-Woge M.A., Martin-Tellez K.S., Gonzalez-Woge R. (2021). Inadequate prediction of postoperative complications in breast cancer surgery: An evaluation of the ACS Surgical Risk Calculator. J Surg Oncol.

[32] Jonczyk M.M., Fisher C.S., Babbitt R. (2021). Surgical predictive model for breast cancer patients assessing acute postoperative complications: the breast cancer surgery risk calculator. Ann Surg Oncol.

[33] Jonczyk M.M., Karamchandani M., Zaccardelli A. (2023). External validation of the breast cancer surgery risk calculator (BCSRc): a predictive model for postoperative complications. Ann Surg Oncol.

[34] Nelson J.A., Chung C.U., Fischer J.P., Kanchwala S.K., Serletti J.M., Wu L.C. (2015). Wound healing complications after autologous breast reconstruction: a model to predict risk. J Plast Reconstr Aesthet Surg.

[35] Park J.W., Jung J.H., Jeon B.J., Mun G.H., Bang S.I., Pyon J.K. (2020). Complications after immediate 2-stage tissue expander/implant breast reconstruction: a deeper look at the second stage. Ann Plast Surg.

[36] Frey J.D., Salibian A.A., Choi M., Karp N.S. (2020). Putting together the pieces: development and validation of a risk-assessment model for nipple-sparing mastectomy. Plast Reconstr Surg.

[37] Mbah C., De Ruyck K., De Schrijver S. (2018). A new approach for modeling patient overall radiosensitivity and predicting multiple toxicity endpoints for breast cancer patients. Acta Oncol.

[38] Kindts I., Defraene G., Petillion S. (2019). Validation of a normal tissue complication probability model for late unfavourable aesthetic outcome after breast-conserving therapy. Acta Oncol.

[39] Meshulam-Derazon S., Yaacobi D.S., Ben-David M.A. (2024). Identifying the variables for oncoplastic reconstruction: preoperative assessment tool for breast conserving treatment. Aesthetic Plast Surg.

[40] Naoum G.E., Ho A.Y., Shui A. (2022). Risk of developing breast reconstruction complications: a machine-learning nomogram for individualized risk estimation with and without postmastectomy radiation therapy. Plast Reconstr Surg.

[41] Cardoso M.J., Cardoso J., Amaral N. (2007). Turning subjective into objective: the BCCT.core software for evaluation of cosmetic results in breast cancer conservative treatment. Breast.

[42] Garbey M., Salmon R., Thanoon D., Bass B.L. (2013). Multiscale modeling and distributed computing to predict cosmesis outcome after a lumpectomy. J Comput Phys.

[43] Vavourakis V., Eiben B., Hipwell J.H., Williams N.R., Keshtgar M., Hawkes D.J. (2016). Multiscale mechano-biological finite element modelling of oncoplastic breast surgery-numerical study towards surgical planning and cosmetic outcome prediction. PLoS One.

[44] Rajula H.S.R., Verlato G., Manchia M., Antonucci N., Fanos V. (2020). Comparison of conventional statistical methods with machine learning in medicine: diagnosis, drug development, and treatment. Medicina (Kaunas).

[45] Soh C.L., Shah V., Arjomandi Rad A. (2022). Present and future of machine learning in breast surgery: systematic review. Br J Surg.

[46] Seth I., Bulloch G., Joseph K., Hunter-Smith D.J., Rozen W.M. (2023). Use of artificial intelligence in the advancement of breast surgery and implications for breast reconstruction: a narrative review. J Clin Med.

[47] Cabitza F., Campagner A., Soares F. (2021). The importance of being external. methodological insights for the external validation of machine learning models in medicine. Comput Methods Programs Biomed.

[48] Collins G.S., Ogundimu E.O., Altman D.G. (2016). Sample size considerations for the external validation of a multivariable prognostic model: a resampling study. Stat Med.

