# Supplementary material for: Prediction models of adverse outcomes following surgery and radiotherapy for breast cancer: a systematic review
Source: ESMO Real World Data Digit Oncol. 2026 Mar 10;11:100690. doi: 10.1016/j.esmorw.2026.100690 (PMC12993883; doi:10.1016/j.esmorw.2026.100690)

**Supplementary Table S1:** Search strategy employed across five databases using different keywords, MeSH terms, and Boolean operators.

| **Database** | **Search strategy** |
| --- | --- |
| PubMed | (quantif*[tiab] OR estimat*[tiab] OR analys*[tiab] OR evaluat*[tiab] OR assess*[tiab] OR measur*[tiab] OR predict*[tiab] OR model*[tiab] OR "Models, Structural"[Mesh] OR tool*[tiab] OR method*[tiab] OR computation*[tiab] OR "Software"[Mesh] OR scal*[tiab] OR scor*[tiab] OR calculat*[tiab] OR “machine learning”[tiab] OR algorithm*[tiab] OR "Algorithms"[Mesh] OR photograph*[tiab] OR "Photography"[Mesh] OR “3?D imag*”[tiab] OR “3?D reconstruction”[tiab] OR "Imaging, Three-Dimensional"[Mesh] OR “risk *"[tiab] OR "Risk Factors"[Mesh]) AND ("breast size"[tiab:~3] OR "breast shape"[tiab:~3] OR "breast volume"[tiab:~3] OR "breast surface"[tiab:~3] OR “breast contour” [tiab:~3] OR "breast atrophy"[tiab:~3] OR "breast morphometric"[tiab:~3] OR "breast morphometrics"[tiab:~3] OR "breast morphometry"[tiab:~3] OR "breast anthropometric"[tiab:~3] OR "breast anthropometrics"[tiab:~3] OR "breast anthropomety"[tiab:~3] OR "breast symmetry"[tiab:~3] OR "breast deformation"[tiab:~3] OR "breast deformity"[tiab:~3] OR "breast skin"[tiab:~3] OR “breast aesthetic”[tiab:~3] OR “breast aesthetics”[tiab:~3] OR “breast esthetic”[tiab:~3] OR “breast esthetics”[tiab:~3] OR “breast cosmesis"[tiab:~3] OR “breast cosmetic"[tiab:~3] OR “breast cosmetics"[tiab:~3] OR "breast radiotoxicity"[tiab:~3] OR "breast radiation toxicity"[tiab:~3]) AND (surgery[tiab] OR "Mastectomy, Segmental"[Mesh] OR reconstruction[tiab] OR "Mammaplasty"[Mesh] OR radiotherapy[tiab] or “Radiotherapy”[Mesh]) AND (“breast cancer”[tiab] OR "Breast Neoplasms"[Mesh]) |
| Medline  (Ovid) | (quantif*.ab,kf,ti. or estimat*.ab,kf,ti. or analys*.ab,kf,ti. or evaluat*.ab,kf,ti. or assess*.ab,kf,ti. or measur*.ab,kf,ti. or predict*.ab,kf,ti. or model*.ab,kf,ti. or exp Models, Structural/ or tool*.ab,kf,ti. or method*.ab,kf,ti. or computation*.ab,kf,ti. or exp Software/ or scal*.ab,kf,ti. or scor*.ab,kf,ti. or calculat.ab,kf,ti. or (machine learning).ab,kf,ti. or algorithm*.ab,kf,ti. or exp Algorithms/ or photograph*.ab,kf,ti. or exp Photography/ or (3?D imag*).ab,kf,ti. or (3?D reconstruction).ab,kf,ti. or exp Imaging, Three-Dimensional/ or (risk *).ab,kf,ti. or exp Risk Factors/) and ((breast adj3 size) or (breast adj3 shape) or (breast adj3 volume) or (breast adj3 surface) or (breast adj3 contour) or (breast adj3 atrophy) or (breast adj3 morphometric) or (breast adj3 morphometrics) or (breast adj3 morphometry) or (breast adj3 anthropometric) or (breast adj3 anthropometrics) or (breast adj3 anthropometry) or (breast adj3 symmetry) or (breast adj3 deformation) or (breast adj3 deformity) or (breast adj3 skin) or (breast adj3 aesthetic) or (breast adj3 aesthetics) or (breast adj3 esthetic) or (breast adj3 esthetics) or (breast adj3 cosmesis) or (breast adj3 cosmetic) or (breast adj3 cosmetics) or (breast ad3 radiotoxicity) or (breast adj3 radiation adj3 toxicity)).ab,kf,ti. and (surgery.ab,kf,ti. or exp Mastectomy, Segmental/ or reconstruction.ab,kf,ti. or exp Mammaplasty/ or radiotherapy.ab,kf,ti. or exp Radiotherapy/) and ((breast cancer).ab,kf,ti. or exp Breast Neoplasms/) |
| Scopus | (TITLE-ABS-KEY (quantif* OR estimat* OR analys* OR evaluat* OR assess* OR measur* OR predict* OR model* OR tool* OR method* OR computation* OR software* OR scal* OR scor* OR calculat* OR "machine learning" OR algorithm* OR photograph* OR "3?D imag*" OR "3?D reconstruction" OR "risk *")) AND (TITLE-ABS-KEY (( breast W/3 size ) OR ( breast W/3 shape ) OR ( breast W/3 volume ) OR ( breast W/3 surface ) OR ( breast W/3 contour ) OR ( breast W/3 atrophy ) OR ( breast W/3 morphometric ) OR ( breast W/3 morphometrics ) OR ( breast W/3 morphometry ) OR ( breast W/3 anthropometric ) OR ( breast W/3 anthropometrics ) OR ( breast W/3 anthropometry ) OR ( breast W/3 symmetry ) OR ( breast W/3 deformation ) OR ( breast W/3 deformity ) OR ( breast W/3 skin ) OR ( breast W/3 aesthetic ) OR ( breast W/3 aesthetics ) OR ( breast W/3 esthetic ) OR ( breast W/3 esthetics ) OR ( breast W/3 cosmesis ) OR ( breast W/3 cosmetic ) OR ( breast W/3 cosmetics ) OR ( breast W/3 radiotoxicity ) OR ( breast W/3 radiation W/3 toxicity ))) AND (TITLE-ABS-KEY ((surgery OR reconstruction OR mammoplasty OR radiotherapy) AND ( breast cancer ))) |
| Web of science | (TS= (quantif* OR estimat* OR analys* OR evaluat* OR assess* OR measur* OR predict* OR model* OR tool* OR method* OR computation* OR software* OR scal* OR scor* OR calculat* OR "machine learning" OR algorithm* OR photograph* OR "3?D imag*" OR "3?D reconstruction" OR "risk*")) AND (TS= (( breast NEAR/3 size ) OR ( breast NEAR/3 shape ) OR ( breast NEAR/3 volume ) OR ( breast NEAR/3 surface ) OR ( breast NEAR/3 contour ) OR ( breast NEAR/3 atrophy ) OR ( breast NEAR/3 morphometric ) OR ( breast NEAR/3 morphometrics ) OR ( breast NEAR/3 morphometry ) OR ( breast NEAR/3 anthropometric ) OR ( breast NEAR/3 anthropometrics ) OR ( breast NEAR/3 anthropometry ) OR ( breast NEAR/3 symmetry ) OR ( breast NEAR/3 deformation ) OR ( breast NEAR/3 deformity ) OR ( breast NEAR/3 skin ) OR ( breast NEAR/3 aesthetic ) OR ( breast NEAR/3 aesthetics ) OR ( breast NEAR/3 esthetic ) OR ( breast NEAR/3 esthetics ) OR ( breast NEAR/3 cosmesis ) OR ( breast NEAR/3 cosmetic ) OR ( breast NEAR/3 cosmetics ) OR ( breast NEAR/3 radiotoxicity ) OR ( breast NEAR/3 radiation NEAR/3 toxicity ))) AND (TS= (surgery OR reconstruction OR mammoplasty OR radiotherapy)) AND (TS= (breast cancer)) |
| CINHAL | ((TI (quantif* or estimat* or analys* or evaluat* or assess* or measur* or predict* or model* or tool* or method* or computation or software or scal* or scor* or calculat* or "machine learning" or algorithm* or photograph* or "3?D imag*" or "3?D reconstruction" or "risk *") or AB (quantif* or estimat* or analys* or evaluat* or assess* or measur* or predict* or model* or tool* or method* or computation or software or scal* or scor* or calculat* or "machine learning" or algorithm* or photograph* or "3?D imag*" or "3?D reconstruction" or "risk *"))) and ((TI ((breast N3 size) or (breast N3 shape) or (breast N3 volume) or (breast N3 surface) or (breast N3 contour) or (breast N3 atrophy) or (breast N3 morphometric) or (breast N3 morphometrics) or (breast N3 morphometry) or (breast N3 anthropometric) or (breast N3 anthropometrics) or (breast N3 anthropometry) or (breast N3 symmetry) or (breast N3 deformation) or (breast N3 deformity) or (breast N3 skin) or (breast N3 aesthetic) or (breast N3 aesthetics) or (breast N3 esthetic) or (breast N3 esthetics) or (breast N3 cosmesis) or (breast N3 cosmetic) or (breast N3 cosmetics) or (breast N3 radiotoxicity) or (breast N3 radiation N3 toxicity))) or (AB ((breast N3 size) or (breast N3 shape) or (breast N3 volume) or (breast N3 surface) or (breast N3 contour) or (breast N3 atrophy) or (breast N3 morphometric) or (breast N3 morphometrics) or (breast N3 morphometry) or (breast N3 anthropometric) or (breast N3 anthropometrics) or (breast N3 anthropometry) or (breast N3 symmetry) or (breast N3 deformation) or (breast N3 deformity) or (breast N3 skin) or (breast N3 aesthetic) or (breast N3 aesthetics) or (breast N3 esthetic) or (breast N3 esthetics) or (breast N3 cosmesis) or (breast N3 cosmetic) or (breast N3 cosmetics) or (breast N3 radiotoxicity) or (breast N3 radiation N3 toxicity)))) and ((TI (surgery or reconstruction or mammoplasty or radiotherapy)) or (AB (surgery or reconstruction or mammoplasty or radiotherapy))) and ((TI (breast cancer)) or (AB (breast cancer))) |

**Supplementary Table S2:** A heatmap illustrating adherence to the TRIPOD+AI checklist items across the model development studies. This illustration provides a summary of reporting quality and transparency across the included studies. Green indicates that the study fully or partially addressed the corresponding item. Red indicates that the item was not addressed.


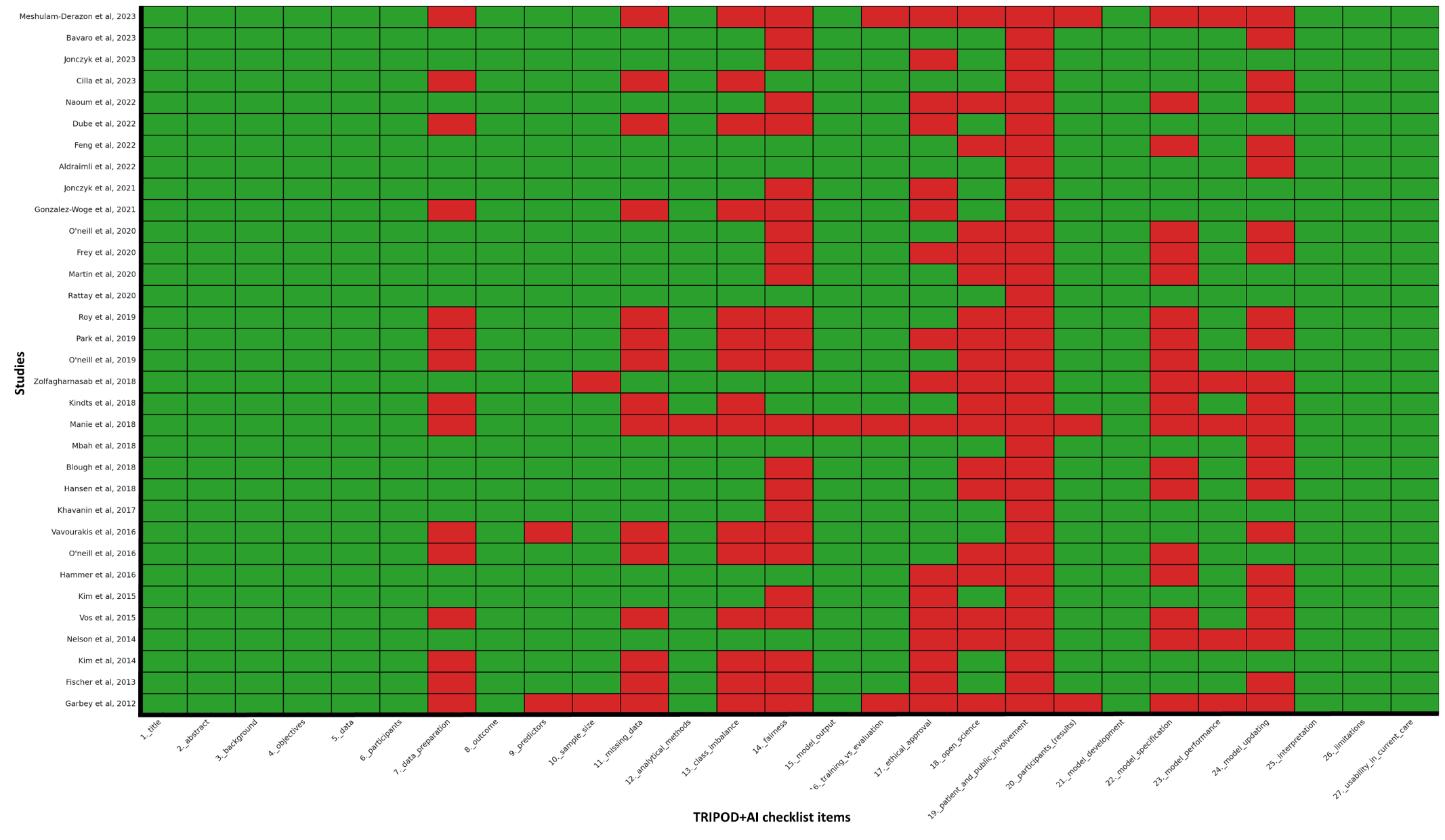

Supplement: Supplementary Tables [file mmc1.docx]
